# Disease course of non-radiographic axial spondyloarthritis: Data from a long-term retrospective observational cohort

**DOI:** 10.1371/journal.pone.0288153

**Published:** 2023-06-30

**Authors:** Oh Chan Kwon, Yong-Gil Kim, Min-Chan Park

**Affiliations:** 1 Division of Rheumatology, Department of Internal Medicine, Yonsei University College of Medicine, Seoul, South Korea; 2 Division of Rheumatology, Department of Internal Medicine, University of Ulsan College of Medicine, Asan Medical Center, Seoul, South Korea; 3 Convergence Medicine Research Center, Asan Institution for Life Science, Asan Medical Center, Seoul, South Korea; Beni-Suef University, EGYPT

## Abstract

**Background:**

Disease course of non-radiographic axial spondyloarthritis (axSpA) has been extensively studied in non-Asian population; however, there are limited data in Asian population. This study aimed to evaluate the long-term disease course of non-radiographic axSpA in Asian patients and identify factors associated with progression to radiographic axSpA.

**Methods:**

In this retrospective observational cohort study, 56 Korean patients newly diagnosed with non-radiographic axSpA between 2006 and 2015 were included. All patients fulfilled the Assessment of SpondyloArthritis international Society classification criteria for axSpA, and did not fulfil the radiological criterion of the 1984 modified New York criteria. Disease course was assessed by the rate of progression to radiographic axSpA. Factors associated with the risk of progression to radiographic axSpA were assessed using multivariable Cox proportional hazard regression analysis.

**Results:**

The mean age at baseline was 31.4±13.3 years, and 37 (66.1%) patients were men. Over a mean observation period of 8.4±3.7 years, 28 (50.0%) patients progressed to radiographic axSpA. In multivariable Cox proportional hazard regression analysis, the presence of syndesmophytes at diagnosis (adjusted hazard ratio [HR]: 4.50, 95% confidence interval [CI]: 1.54–13.15, p = 0.006) and active sacroiliitis on magnetic resonance imaging (MRI) at diagnosis (adjusted HR: 5.88, 95% CI: 2.05–16.82, p = 0.001) were significantly associated with a higher risk of progression to radiographic axSpA, whereas longer exposure to tumor necrosis factor inhibitors (TNFis) was significantly associated with a lower risk of progression to radiographic axSpA (adjusted HR: 0.89, 95% CI: 0.80–0.98, p = 0.022).

**Conclusion:**

During long-term follow-up, a substantial proportion of Asian patients with non-radiographic axSpA progressed to radiographic axSpA. The presence of syndesmophytes and active sacroiliitis on MRI at the time of non-radiographic axSpA diagnosis were associated with a higher risk of progression to radiographic axSpA, while longer exposure to TNFis was associated with a lower risk of progression to radiographic axSpA.

## Introduction

Axial spondyloarthritis (axSpA) is a chronic inflammatory disease that primarily affects the axial skeleton leading to bony ankylosis of the sacroiliac joints and spine [1, 2]. The spectrum of axSpA encompasses both non-radiographic and radiographic axSpA, which are distinguished based on the structural damage of the sacroiliac joints on radiographs [1, 3]. Non-radiographic and radiographic axSpA have comparable disease burden, and similar responses to biologic agents, including tumor necrosis factor inhibitors (TNFis) and interleukin-17A inhibitors (IL-17is) [4–8]. Differences between the two axSpA subsets have also been noted. Compared with radiographic axSpA, non-radiographic axSpA has a higher proportion of females and a lower rate of syndesmophyte formation over time [3].

Regarding disease course, studies have shown that 10–40% of patients with non-radiographic axSpA progress to radiographic axSpA over a period of 2–10 years [9–11]. While some patients with non-radiographic axSpA progress to radiographic axSpA over time, others do not and remain as non-radiographic axSpA [12]. Moreover, some patients with non-radiographic axSpA might even remit in the course of the disease [12, 13]. Given the varying disease course across patients, studies have assessed factors associated with progression from non-radiographic axSpA to radiographic axSpA: active sacroiliitis on magnetic resonance imaging (MRI), smoking, human leukocyte antigen (HLA)-B27 positivity, and elevated C-reactive protein (CRP) are suggested as factors associated with progression to radiographic axSpA [10, 11, 14, 15]. Active sacroiliitis on MRI is associated with 2- to 50-fold higher risk of progression [11, 14, 15]. Smoking and HLA-B27 positivity are associated with 3.3-fold and 12.6-fold higher risk of progression, respectively [14]. Elevated CRP is associated with 3.65-fold higher risk of progression [10].

Although the disease course of non-radiographic axSpA has been extensively studied, the majority of the studies have been conducted in non-Asian countries [10, 11, 14–17]. Clinical features of non-radiographic axSpA vary among different ethnic populations. A cross-sectional study reported that Asian patients with non-radiographic axSpA are predominantly male, younger at disease onset, and have a higher positivity rate of HLA-B27 as compared with patients with non-radiographic axSpA from other regions [18]. The difference in clinical characteristics among different populations might be explained by varying distributions of HLA and other genetic factors [18]. Hence, it is likely that disease course of non-radiographic axSpA in Asian patients may also differ from that of non-Asian patients. However, data on disease course of non-radiographic axSpA in Asian patients are limited. A study from Korea described the disease course of non-radiographic axSpA in Asian patients. However, the observation duration was relatively short (median: 4 years) [19]. Data on disease course of Asian patients with non-radiographic axSpA over a longer-term follow-up are still lacking.

We hypothesized that the long-term disease course of non-radiographic axSpA in Asian patients may differ from that in non-Asian patients. To better understand the disease course of non-radiographic axSpA in Asian patients, we described the rate of progression to radiographic axSpA in Asian patients with non-radiographic axSpA during a long-term observation period. We also aimed to identify factors associated with progression.

## Materials and methods

### Study design, setting, sample size

We conducted a retrospective observational cohort study. Data were retrospectively collected from electronic medical records of the patients at a tertiary referral hospital in Seoul, South Korea. The sample size was determined according to the inclusion and exclusion criteria as follows. Inclusion criteria: (i) fulfilling the Assessment of SpondyloArthritis international Society (ASAS) classification criteria for axSpA [20], and (ii) not fulfilling the radiological criterion of the 1984 modified New York criteria [21]. Exclusion criterion was follow up of less than a year.

### Participants

In this study, 61 patients newly diagnosed with non-radiographic axSpA at a tertiary referral hospital in Seoul, South Korea, between January 2006 and December 2015, were retrospectively selected for inclusion in the study. All patients fulfilled the ASAS classification criteria for axSpA [20], and did not fulfil the radiological criterion of the 1984 modified New York criteria [21]. In all patients, radiographs were followed up every 2–3 years. The retrospective observation period was from the date of diagnosis of non-radiographic axSpA to the date when the last radiographs were taken (until December 2021), or the date when progression to radiographic axSpA was first detected, whichever occurred first. Patients who were followed up for less than a year (n = 5) were excluded. The remaining 56 patients with non-radiographic axSpA were included for the final analysis.

### Issue of interest

The data analysis included covariates at the time of non-radiographic axSpA diagnosis, such as age, sex, symptom duration (time from symptom onset to diagnosis), smoking status (current smoker, yes or no), body mass index, HLA-B27 positivity, peripheral symptoms, uveitis, psoriasis, inflammatory bowel diseases, erythrocyte sedimentation rate (ESR), CRP level, sacroiliitis sum score, active sacroiliitis on MRI, and the presence of syndesmophytes. Based on the radiograph, sacroiliitis of each sacroiliac joint was graded according to the modified New York criteria [21]. The sacroiliitis sum score was calculated as the sum of the right and left sacroiliitis grades [10, 22]. The presence of syndesmophytes was determined from the cervical and lumbar spine radiographs. Radiographs were interpreted at the time of imaging by expert musculoskeletal radiologists.

The use of medications, such as non-steroidal anti-inflammatory drugs (NSAIDs), sulfasalazine, methotrexate, TNFis, and IL-17is, during the observation period was taken into account. For NSAIDs, the ASAS NSAID intake score [23] was calculated. For TNFis and IL-17is, exposure durations were assessed.

### Comparison

The patients were categorized into those who did not progress to radiographic axSpA and those who progressed to radiographic axSpA. Covariates mentioned above were compared between the two groups.

### Ethics and end point

The Institutional Review Board (IRB) of the Gangnam Severance Hospital, Seoul, South Korea, approved this study (IRB No: 3-2018-0283). Owing to the retrospective nature of this study, the requirement for informed consent was waived.

The study end point was progression to radiographic axSpA, which was determined based on follow-up radiographs of the sacroiliac joints in each patient. Patients, who newly fulfilled the radiological criterion of the 1984 modified New York criteria [21], were considered to have progressed to radiographic axSpA.

### Statistical analysis

Continuous variables following normal or non-normal distributions were expressed as means ± standard deviation or medians (interquartile range), respectively. Categorical variables were expressed as numbers (%). For comparison between two patient groups, independent two sample t-test or Mann-Whitney U test was used for continuous variables following normal or non-normal distributions, respectively. χ2 test or Fisher’s exact test was used for comparison of categorical variables. Cox proportional hazard regression analysis was conducted to identify factors associated with progression to radiographic axSpA. Univariable analysis was initially performed. Factors with p < 0.05 in the univariable analysis were included in the multivariable analysis. Statistical significance was set at p < 0.05. All analyses were performed using IBM SPSS Statistics for Windows, version 25.0 (IBM Corp., Armonk, NY, USA).

## Results

### Patient characteristics and the rate of progression to radiographic axSpA

In this study, data from 56 patients with non-radiographic axSpA were analyzed. The mean observation duration of the patients was 8.4 ± 3.7 years. The mean age of the patients at baseline was 31.4 ± 13.3 years, and 37 (66.1%) patients were men. The median symptom duration was 2.3 (1.8–3.5) years. At baseline radiographs, the median value of the sacroiliitis sum score was 1.0 (0.0–2.0), and syndesmophytes were present in 8 (14.3%) patients. Active sacroiliitis was observed on MRI in 36 (64.3%) patients. Regarding medications, TNFis and IL-17is were used in 28 (50.0%) and 2 (3.6) patients, respectively. For patients who were exposed to TNFis, the median duration of TNFi exposure was 4.9 (3.3–9.7) years. Two patients were exposed to IL-17is, with exposure durations of 1.3 and 3.2 years, respectively. Detailed patient characteristics are summarized in Table 1. Of the 56 patients with non-radiographic axSpA, 28 (50.0%) patients had progressed to radiographic axSpA over 8.4 ± 3.7 years of follow-up.

**Table 1:** Baseline characteristics of 56 patients with non-radiographic axial spondyloarthritis.

	N = 56
Age, years, mean ± SD	31.4 ± 13.3
Male sex, n (%)	37 (66.1)
Symptom duration, years, median (IQR)	2.3 (1.8–3.5)
Current smoker, n (%)	3 (5.4)
BMI, kg/m^2^, mean ± SD	23.8 ± 3.4
HLA-B27 positive n (%)	43 (76.8)
Peripheral symptoms, n (%)	38 (67.9)
Uveitis, n (%)	11 (19.6)
Psoriasis, n (%)	2 (3.6)
IBD, n (%)	2 (3.6)
ESR, mm/h, median (IQR)	17.0 (8.0–39.8)
Elevated ESR, n (%)	27 (48.2)
CRP, mg/L, median (IQR)	3.0 (1.2–14.5)
Elevated CRP, n (%)	21 (37.5)
Sacroiliitis sum score, median (IQR)	1.0 (0.0–2.0)
Presence of syndesmophytes, n (%)	8 (14.3)
Active sacroiliitis on MRI, n (%)	36 (64.3)
Medications used between radiographs	
Use of NSAIDs, n (%)	56 (100.0)
NSAID intake score, mean ± SD	50.3 ± 25.3
Use of sulfasalazine, n (%)	37 (66.1)
Use of methotrexate, n (%)	14 (25.0)
Use of TNFi, n (%)	28 (50.0)
Exposure duration of TNFi^a^, years, median (IQR)	4.9 (3.3–9.7)
Use of IL-17i, n (%)	2 (3.6)
Progression to radiographic axSpA, n (%)	28 (50.0)

^a^Patients without TNFi exposure were excluded.

BMI, body mass index; CRP, C-reactive protein; ESR, erythrocyte sedimentation rate; HLA, human leukocyte antigen; IBD, inflammatory bowel disease; IL-17i, interleukin-17A inhibitor; IQR, interquartile range; MRI, magnetic resonance imaging; NSAIDs, non-steroidal anti-inflammatory drugs; TNFi, tumor necrosis factor inhibitor; SD, standard deviation.

### Comparison of patients who did not progress to radiographic axSpA and those who did

Comparison of the baseline characteristics between the two groups are reported in Table 2. Compared with patients who did not progress to radiographic axSpA, those who progressed to radiographic axSpA more frequently had syndesmophytes at baseline (3.6% vs. 25.0%, p = 0.022), and active sacroiliitis on MRI at baseline (50.0% vs. 78.6%, p = 0.026). There were no significant differences in other covariates between the two groups.

**Table 2:** Comparison of the baseline characteristics between patients who did not progress to radiographic axial spondyloarthritis and those who did.

	Not progressed (N = 28)	Progressed (N = 28)	P value
Age, years, mean ± SD	31.5 ± 13.6	31.3 ± 13.2	0.953
Male sex, n (%)	20 (71.4)	17 (60.7)	0.397
Symptom duration, years, median (IQR)	2.1 (1.8–2.5)	2.5 (1.7–3.6)	0.232
Current smoker, n (%)	2 (7.1)	1 (3.6)	>0.999
BMI, kg/m^2^, mean ± SD	24.2 ± 3.7	23.5 ± 3.0	0.498
HLA-B27 positive, n (%)	21 (75.0)	22 (78.6)	0.752
Peripheral symptoms, n (%)	21 (75.0)	17 (60.7)	0.252
Uveitis, n (%)	6 (21.4)	5 (17.9)	0.737
Psoriasis, n (%)	2 (7.1)	0 (0.0)	0.491
IBD, n (%)	1 (3.6)	1 (3.6)	>0.999
ESR, mm/h, median (IQR)	15.0 (6.0–37.0)	24.5 (9.5–47.8)	0.207
Elevated ESR, n (%)	11 (39.3)	16 (57.1)	0.181
CRP, mg/L, median (IQR)	2.7 (0.4–11.9)	5.3 (1.3–23.0)	0.241
Elevated CRP, n (%)	9 (32.1)	12 (42.9)	0.408
Sacroiliitis sum score, median (IQR)	2.0 (0.0–2.0)	1.0 (0.0–2.0)	0.478
Presence of syndesmophytes, n (%)	1 (3.6)	7 (25.0)	0.022
Active sacroiliitis on MRI, n (%)	14 (50.0)	22 (78.6)	0.026
Medications used between radiographs			
Use of NSAIDs, n (%)	28 (100.0)	28 (100.0)	N/A
NSAID intake score, mean ± SD	54.6 ± 26.1	46.0 ± 24.0	0.202
Use of sulfasalazine, n (%)	21 (75.0)	16 (57.1)	0.158
Use of methotrexate, n (%)	5 (17.9)	9 (32.1)	0.217
Use of TNFi, n (%)	16 (57.1)	12 (42.9)	0.285
Exposure duration of TNFi^a^, years, median (IQR)	5.0 (3.6–10.7)	4.4 (1.9–7.8)	0.371
Use of IL-17i, n (%)	2 (7.1)	0 (0.0)	0.491

^a^Patients without TNFi exposure were excluded.

BMI, body mass index; CRP, C-reactive protein; ESR, erythrocyte sedimentation rate; HLA, human leukocyte antigen; IBD, inflammatory bowel disease; IL-17i, interleukin-17A inhibitor; IQR, interquartile range; MRI, magnetic resonance imaging; NSAIDs, non-steroidal anti-inflammatory drugs; TNFi, tumor necrosis factor inhibitor; SD, standard deviation.

### Factors associated with progression to radiographic axSpA

The results of the Cox proportional hazard regression analysis are shown in Table 3. Univariable analysis showed that longer symptom duration (unadjusted hazard ratio [HR]: 1.14, 95% confidence interval [CI]: 1.02–1.27, p = 0.018), presence of syndesmophytes at baseline (unadjusted HR: 4.70, 95% CI: 1.89–11.70, p = 0.001), and active sacroiliitis on MRI at baseline (unadjusted HR: 3.97, 95% CI: 1.54–10.23, p = 0.004) were significantly associated with a higher risk of progression to radiographic axSpA. In contrast, longer duration of exposure to TNFis (unadjusted HR: 0.88, 95% CI: 0.80–0.98, p = 0.014) was associated with a lower risk of progression to radiographic axSpA. However, when analyzed as a categorical variable (yes vs. no), TNFi usage (unadjusted HR: 0.67, 95% CI: 0.31–1.44, p = 0.303) was not significantly associated with a lower risk of progression to radiographic axSpA. In the multivariable analysis, symptom duration (adjusted HR: 1.06, 95% CI: 0.95–1.19, p = 0.276) lost statistical significance, while the presence of syndesmophytes at baseline (adjusted HR: 4.50, 95% CI: 1.54–13.15, p = 0.006), active sacroiliitis on MRI at baseline (adjusted HR: 5.88, 95% CI: 2.05–16.82, p = 0.001), and TNFi exposure duration (adjusted HR: 0.89, 95% CI: 0.80–0.98, p = 0.022) remained as statistically significant.

**Table 3:** Factors associated with progression to radiographic axial SpA.

	Univariable analysis		Multivariable analysis	
	Unadjusted HR (95% CI)	P value	Adjusted HR (95% CI)	P value
Age	1.01 (0.98–1.04)	0.604		
Male sex	0.72 (0.33–1.56)	0.400		
Symptom duration	1.14 (1.02–1.27)	0.018	1.06 (0.95–1.19)	0.276
Current smoker	0.79 (0.10–5.94)	0.817		
BMI, kg/m^2^	0.90 (0.78–1.03)	0.117		
HLA-B27 positive	1.22 (0.49–3.05)	0.673		
Peripheral symptoms	0.73 (0.34–1.58)	0.423		
Uveitis	0.33 (0.09–1.14)	0.079		
Psoriasis	0.05 (0.00–7642.29)	0.617		
IBD	0.68 (0.09–5.07)	0.706		
ESR	1.00 (0.99–1.02)	0.532		
Elevated ESR	1.62 (0.75–3.51)	0.222		
CRP	1.00 (0.98–1.01)	0.572		
Elevated CRP	1.45 (0.67–3.15)	0.345		
Sacroiliitis sum score	0.76 (0.51–1.15)	0.191		
Presence of syndesmophytes	4.70 (1.89–11.70)	0.001	4.50 (1.54–13.15)	0.006
Active sacroiliitis on MRI	3.97 (1.54–10.23)	0.004	5.88 (2.05–16.82)	0.001
NSAID intake score	1.00 (0.99–1.02)	0.817		
Use of sulfasalazine	0.53 (0.25–1.14)	0.106		
Use of methotrexate	1.22 (0.53–2.82)	0.639		
Use of TNFi	0.67 (0.31–1.44)	0.303		
Exposure duration of TNFi	0.88 (0.80–0.98)	0.014	0.89 (0.80–0.98)	0.022
Use of IL-17i	0.05 (0.00–41119.00)	0.662		

BMI, body mass index; CI, confidence interval; CRP, C-reactive protein; ESR, erythrocyte sedimentation rate; HLA, human leukocyte antigen; HR, hazard ratio; IBD, inflammatory bowel disease; IL-17i, interleukin-17A inhibitor; MRI, magnetic resonance imaging; NSAIDs, non-steroidal anti-inflammatory drugs; TNFi, tumor necrosis factor inhibitor.; axSpA, axial spondyloarthritis

## Discussion

In this long-term observational study, we assessed the rate of progression to radiographic axSpA in Asian patients with non-radiographic axSpA. Progression to radiographic axSpA occurred in 50% of the patients with non-radiographic axSpA over a mean observation period of 8.4 years. We also found that presence of syndesmophytes and active sacroiliitis on MRI at diagnosis of non-radiographic axSpA are associated with a higher risk of progression to radiographic axSpA, whereas longer duration of TNFi exposure is associated with a lower risk of progression to radiographic axSpA. These findings are meaningful as they provide us better knowledge regarding the long-term disease course of non-radiographic axSpA in Asian patients, which has not been extensively studied as compared with that in non-Asian patients.

Although direct statistical comparison cannot be made, the progression rate of 50% over a mean observation period of 8.4 years is relatively higher than the rates reported by studies in non-Asian countries (10–40% over an observation period of 2–10 years) [9–11]. The higher progression rate in our study could be attributed to differences in clinical characteristics between Asian and non-Asian patients with non-radiographic axSpA. A previous study reported that Asian patients with non-radiographic axSpA were more frequently male (75.9% vs. 47.1%, p < 0.001), younger age at diagnosis (27.2 [21.1–39.6] years vs. 34.5 [27.7–41.7] years, p < 0.001), and had higher HLA-B27 positivity rate (90.6% vs. 61.9%, p < 0.001) than non-Asian patients with non-radiographic axSpA [18]. Similarly, patients in our study were predominantly male (66.1%), relatively young (mean age: 31.4 years), and had a high positivity rate of HLA-B27 (76.8%). These differences in characteristics between Asian and non-Asian patients with non-radiographic axSpA might have led to a more progressive disease course in Asian patients.

In the analysis of factors associated with progression to radiographic axSpA, the presence of syndesmophytes at diagnosis was associated with a 4.5-fold increase in risk of progression to radiographic axSpA (adjusted HR: 4.50). Studies have shown that the presence of syndesmophytes at diagnosis in patients with axSpA is associated with a 6- to 18-fold increased risk of radiographic spinal progression [24–26]. Our results suggest that the presence of syndesmophytes at diagnosis is also associated with a higher risk progression in the structural damage of the sacroiliac joints.

We also observed that active sacroiliitis on MRI at diagnosis is associated with approximately 6-fold increase in risk of progression to radiographic axSpA (adjusted HR: 5.88). This finding is consistent with studies from non-Asian countries, which reported active sacroiliitis on MRI as a factor associated with progression to radiographic axSpA [14–16]. On the other hand, the sacroiliitis sum score at diagnosis was not associated with the risk of progression to radiographic axSpA. Based on our findings, the presence of active inflammation in the sacroiliac joints at diagnosis, rather than structural change in the sacroiliac joints at diagnosis, seems to be a more reliable risk factor for progression to radiographic axSpA.

In our study, another factor associated with the risk of progression to radiographic axSpA was TNFi exposure duration. Longer TNFi exposure was associated with a lower risk of progression to radiographic axSpA (adjusted HR: 0.89), accounting for a 11% decrease in risk of progression per 1-year of exposure to TNFis. This finding is in line with studies suggesting that the use of TNFis may impede the progression of the radiographic structural damage of the sacroiliac joints [27–29]. However, in our analysis, when TNFi usage (yes vs. no) was analyzed without considering exposure duration, no significant association was observed between TNFi usage and the risk of progression to radiographic axSpA. This finding suggests that TNFi exposure duration is more important than TNFi usage itself, in terms of lowering the risk of progression to radiographic axSpA. Longer exposure to TNFis in patients with non-radiographic axSpA could contribute to a higher chance of remaining in a non-progressive disease.

This study has some limitations. First, as a retrospective study, bias resulting from unmeasured confounders could not be fully excluded. Second, the number of patients was relatively small. Only two patients were exposed to IL-17is, and we were unable to analyze whether the exposure duration to IL-17is was associated with the risk of progression to radiographic axSpA. As there are currently very limited data on the effect of IL-17is on the progression from non-radiographic axSpA to radiographic axSpA, further studies are required. Third, we lacked data on disease activity indices, such as the Bath Ankylosing Spondylitis Disease Activity Index [30] and the Ankylosing Spondylitis Disease Activity Score [31] at diagnosis of non-radiographic axSpA. Active sacroiliitis on MRI, ESR, and CRP were considered as proxies of disease activity instead. Despite these limitations, our study is clinically significant as it provides important data on long-term disease course of non-radiographic axSpA and factors associated with progression to radiographic axSpA, specifically in Asian patients, which have been poorly studied to date.

## Conclusion

In conclusion, in our cohort of Korean patients with non-radiographic axSpA, 50% of the patients progressed to radiographic axSpA over a mean observation period of 8.4 years, which is relatively higher than that in studies of patients from non-Asian countries. The presence of syndesmophytes and active sacroiliitis on MRI at diagnosis of non-radiographic axSpA were associated with a higher risk of progression to radiographic axSpA, whereas longer exposure to TNFis was associated with a lower risk of progression to radiographic axSpA. These data improve our understanding of the long-term disease course of non-radiographic axSpA as well as factors associated with disease progression in Asian patients.

## Supporting information

S1 Data (XLSX)
